# Controlled Positioning of Cells in Biomaterials—Approaches Towards 3D Tissue Printing

**DOI:** 10.3390/jfb2030119

**Published:** 2011-08-04

**Authors:** Silke Wüst, Ralph Müller, Sandra Hofmann

**Affiliations:** Institute for Biomechanics, ETH Zurich, 8093 Zurich, Switzerland; E-Mails: siwuest@ethz.ch (S.W.); ram@ethz.ch (R.M.)

**Keywords:** tissue engineering, bioprinting, 3D scaffolds, hydrogel, tissue regeneration

## Abstract

Current tissue engineering techniques have various drawbacks: they often incorporate uncontrolled and imprecise scaffold geometries, whereas the current conventional cell seeding techniques result mostly in random cell placement rather than uniform cell distribution. For the successful reconstruction of deficient tissue, new material engineering approaches have to be considered to overcome current limitations. An emerging method to produce complex biological products including cells or extracellular matrices in a controlled manner is a process called bioprinting or biofabrication, which effectively uses principles of rapid prototyping combined with cell-loaded biomaterials, typically hydrogels. 3D tissue printing is an approach to manufacture functional tissue layer-by-layer that could be transplanted *in vivo* after production. This method is especially advantageous for stem cells since a controlled environment can be created to influence cell growth and differentiation. Using printed tissue for biotechnological and pharmacological needs like *in vitro* drug-testing may lead to a revolution in the pharmaceutical industry since animal models could be partially replaced by biofabricated tissues mimicking human physiology and pathology. This would not only be a major advancement concerning rising ethical issues but would also have a measureable impact on economical aspects in this industry of today, where animal studies are very labor-intensive and therefore costly. In this review, current controlled material and cell positioning techniques are introduced highlighting approaches towards 3D tissue printing.

## 1. Introduction—the Beginning of a Printing Area in Tissue Engineering

The most common application for printing is reproducing text and images usually by writing with ink on paper. This printing area resulted from the invention of the printing press by Johannes Gutenberg around 1440. In the late 1980s, rapid prototyping (RP) appeared on the market making use of the printing technology for expansion into three dimensions (3D). Material was added layer-by-layer with this manufacturing technique mainly for producing models and prototype parts. During the last decade the printing process has been adapted for a large range of applications such as printing quality parts of small numbers, transistor circuits [1] or biological material including protein and cells [2]. The advantage of printing technology is that two-dimensional (2D) patterns can be predesigned for example in PowerPoint and 3D constructs with computer-aided design (CAD) tools and reprinted without limitations. In the range of the printer's resolution, which is nowadays between 1 μm and several 100 μm depending on the printer type, the printing material still named “ink” can be deposited in a controlled fashion and according to the virtual design.

Classical tissue engineering refers to seeding isolated cells on solid scaffolds as introduced by Langer and Vacanti almost two decades ago [3] and is still a cutting-edge technology [4]. Biomaterials provide ideally biocompatible and degradable properties for scaffolding and generating cell-scaffold constructs demonstrate promising alternatives for autologous grafting and organ replacement. 3D scaffolds carry the function of the not-yet existing extracellular matrix (ECM) until cells cultured on the constructs produce their own functional matrix. Scaffold properties are of great importance since it is known that many cell types, especially stem cells, are sensitive to the intrinsic properties of ECM, its proteins, substrate surface chemistry, substrate stiffness, chemotransport and soluble signals [5]. The material chemistry, stiffness, porosity, pore size and geometry strongly influence how a cell “feels” and reacts to the scaffold [6,7,8,9,10]. Many different scaffold manufacturing techniques such as salt-leaching [11], porogen melting [12], gas foaming [13,14], electrospinning [15], fiber deposition [16], molding [17] and freeze-drying [18] have been investigated in the past [19,20]. All these conventional techniques cannot avoid heterogeneities in scaffold pore size, porosity, pore interconnectivity and random non-precise scaffold geometries, which makes it complicated to draw conclusions from experiments that investigate the effect of scaffold properties on newly formed tissues [21]. Other limitations of the conventional tissue engineering approach arise in investigating microenvironmental cues of cell-cell and cell-scaffold interactions. With conventional cell seeding techniques such as pipetting cell suspensions on scaffolds by hand, cells cannot be placed in defined locations, which makes it almost impossible to seed various different cell types in special patterns on the scaffold. Controlled positioning of cells on the scaffold would be needed, for example, to determine stresses experienced by the cells at a certain position and also their response to external mechanical forces such as substrate strain and fluid flow. Also, when considering organs the various cell types are not randomly mixed within the construct but specifically arranged according to the need and function, for example endothelial cells (ECs) are aligned to form vessels, or osteoblasts forming mineralized clusters. To address these questions there is great interest in generating not only complex and structurally organized constructs, but also functional implants *in vitro* mimicking physiological tissue [22].

Biofabrication is an emerging technology referring to “manufacture complex 3D tissues and organs by printing or 3D fabrication technologies to overcome the limitations of conventional tissue engineering methods” [23]. It allows cells to be positioned in a controlled way in and together with biomaterials. The fabrication technique is based on RP, which stands for techniques reading in data from CAD drawings and building the 3D objects layer-by-layer according to the virtual design [24]. Biofabrication includes various techniques such as bioprinting, bioplotting, inkjet printing and stereolithography. It is defined as the production of complex living and non-living biological products by placing proteins, peptides, DNA, cells, hormones or ECM molecules together with biomaterials [4,25]. Two main applications have been aimed at by using RP approaches in tissue engineering so far. The patient-specific application is to fill a defect with a functional biofabricated construct of the same shape as the tissue-to-be-replaced. The defect can be measured by computed tomography (CT) or magnetic resonance imaging (MRI) to assess its geometry. This data set is then converted into a CAD model. The information will be transferred to the printer for automatically manufacturing 3D objects [24]. The other more general aspect of biofabrication addresses basic research *in vitro* for studying intrinsic tissue properties or using it for drug-testing in pharmacology. Constructs consisting of different scaffold types such as solid or gel scaffolds as well as various cell patterns can be manufactured with the 3D biofabrication tool to investigate microenvironmental cues. Solid scaffolds of different geometries and complex architecture can be designed and fabricated with defined pore size and interconnectivity [26,27,28]. This controlled fabrication technique can be used to optimize channels for culture medium access throughout the whole scaffold, considering the diffusion limitation of oxygen, nutrition supply and waste removal of about 150 μm [29]. Due to their partly cell-unfriendly manufacturing conditions such as high temperatures or solvents, solid scaffolds cannot be used to print cells and scaffold material in parallel. In the case of cell encapsulation a hydrogel construct is needed. Hydrogels are printed in a liquid-like state and can be subsequently treated for gelling to provide stable 3D architecture during culture [30]. With this procedure, only reduced control level and thus less possible complexity of scaffold architecture can be achieved. Considering again the importance of nutrient supply, researchers started printing vascular-like structures consisting of ECs in defined locations within the hydrogel [31], instead of optimizing channel geometries for medium access as in solid scaffolds. Cells either seeded onto solid scaffolds and attached to the scaffold walls or encapsulated into hydrogels need an environment which is porous enough to allow cell culture medium to enter and support them with nutrients. Printing various living cells within 3D constructs in determined locations next to each other is a relatively new procedure with the first results published in 2004 [32,33]. It is a highly challenging approach, but enormously suitable for mimicking the *in vivo* situation. After printing, the cell arrangement has to be incubated in a physiological environment in order to accelerate cell adhesion, proliferation and differentiation and to become a functional tissue [23].

In this review, the application of 3D bioprinting technology to position cells encapsulated in hydrogels in a controlled way at predefined locations is presented. It has been shown that polymeric hydrogels are a very suitable form for the biofabrication of living cells in 3D. The highly hydrated polymer network prevents cells from drying and from high shear forces during the fabrication process.The hydrogel can act as a supportive 3D environment for cell attachment, proliferation and differentiation and can be enhanced with biomimetic and ECM components which provide biological cues to direct new tissue formation [23]. For example, Fedorovich *et al.* embedded multipotent stromal cells into Lutrol hydrogel, printed the mixture with a bioplotter, photo-crosslinked the hydrogel and showed that the printed osteogenic progenitor cells differentiated along the osteogenic lineage [34]. Whereas liquid ink would spread, hydrogels can be induced to solidify and stabilize the construct. Various gelation techniques have been applied in different tissue engineering studies with or without 3D biofabrication such as photo-, thermo- or chemical gelation, all based on sol-gel reactions. To achieve the crucial task of controlled cell placement in a hydrogel matrix, the following requirements have to be fulfilled: The hydrogel must be formed immediately after ejection of gel precursor ink, for example by printing the hydrogel into an appropriate crosslinker solution. The gel precursor must have a low viscosity for easy ejection by the biofabrication device, whereas the resulting gel has to be stable enough to maintain 3D architectures. The gelation process needs to be cell compatible. The fabrication has to be performed under biological conditions, *i.e.*, the process is not detrimental to the cells and does not modify cellular functions. A variety of hydrogels with different properties has already been used for tissue engineering in general and in 3D biofabrication [35].

This review will point out ongoing studies and possible applications using 3D biofabrication methods for tissue engineering *in vitro* and the importance of this promising field. Various existing 3D bioprinting and biofabrication techniques have been already published previously [4,27,36] and thus will be only briefly discussed. The focus herein is the application for tissue engineering cellular constructs especially emphasizing the applicability and potential for stem cells.

## 2. 2D Biopatterning

A first step towards 3D biofabrication was the 2D placing of cells onto solid substrates, also known as 2D cell patterning or biopatterning. Research groups came up with various methods of how to arrange different types of cells in a predefined pattern next to each other such as photolithography, microstamping, microfluidic patterning, photopatterning and electropatterning [37,38,39,40,41]. The idea of using microfabrication tools for investigating biological phenomena arose when it became obvious that cell fate and function strongly depended on the surface they were cultured on [42]. The first approach, guiding cells with laser light onto a special location on the target, was performed by Odde *et al.* in 1999 [38,43]. Spinal cord cells were successfully guided through culture medium and deposited as small clusters on a glass surface. The cells remained viable and developed normal-appearing neurites after exposure to the laser light. Boland was a pioneer in adapting inkjet printer heads to print the living cells in a predefined pattern. In his first attempts he used a single line of ECs and smooth muscle cells (SMCs) [44]. He was also able to show cell survival and attachment after printing, which is the minimal requirement for the successful introduction of a new technique. In this review, only 2D cell patterning techniques which are expandable for 3D applications are discussed. These methods are presented in the following paragraph, discussing advantages and disadvantages of the various techniques. The broad range of applications for 2D cell patterning is highlighted by various examples listed in Table 1; applications for 3D will be discussed in Section 5.

**Table 1:** Examples of 2D cell patterning applications (LGDW = Laser-Guided Direct Writing; MAPLE DW = Matrix Assisted Pulsed Laser Evaporation Direct Write; BioLP = Biological Laser Printing; LAB = Laser Assisted Bioprinting; ECs = endothelial cells; SMCs = smooth muscle cells; MSCs = mesenchymal stem cells; PVA = polyvinyl alcohol).

**Technology**	**Hydrogel**	**Cells**	**Fabrication specifications**	**References**
***Laser-based cell patterning***				
LGDW	Culture medium	Embryonic chick spinal cord cells	Ø Nozzle: 30–100 mu;mpushing force of cells: 11.4 μm/s; deposition rate: 2.5 cells/min	[38,43]
MAPLE DW	Matrigel	Pluripotent cells, osteoblasts, cardiac cells, embryonal carcinoma cells	<10 μm resolution; laser spot size: 10–100 μm; 10 μm film thickness	[45,46]
BioLP	Matrigel	Osteosarcoma cells	Single cell resolution (μm range); laser focal spot size: 100μm; deposition volumes: 4.5–230 pL; Ødroplet: 70–260 μm; coated layer: 50μm	[47,48,49]
	Alginate	ECs	Ø Droplets: 70 μm containing 5–7 living cells	[50]
LAB	Matrigel, fibrin hydrogel	Carcinoma cells, ECs	Ø Droplets: 40–70 μm	[51]
***Inkjet printing***				
Cell printer (thermally + piezo-based)	Matrigel, collagen, K-70; fibrin	Mammalian cells, proteins, ECs, SMCs	Ø Nozzle: 300 μm; droplet: 130 pL	[2,44,52]
	Collagen substrate	MSCs, cancer cells	Ø Nozzle: 80μm; printing resolution: 84.7 μm; max speed: 8 mm/s; sample size:∼2.4 mm	[53]
Electrostatic inkjet	Alginate; PVA as viscosity enhancer; Culture medium	HeLa cells, ECs	12 nozzles; droplet: 1–100 pL (size droplet ≈ size of cell); Ø printed dots w/o cells: 25–30m; 0–4 cells per dot; Ø printed dots with cells: 85–240 μm; resolution: 0.2 μm;repeatability: ±4 μm	[54,55]
Piezoelectric inkjet	Culture Medium	Fibroblasts	Ø Droplets: 40 μm	[56]
	Agarose substrate	Escherichia coli	Droplet size: 37 ± 0.3μm; resolution: 50–100 μm	[57]

### 2.1. Laser-Based Cell Patterning

Placing cells into special patterns with the help of laser light has been one of the first methods to approach 2D cell patterning. The various different methods arose mainly by use of laser light to move cells. Laser-Guided Direct Writing (LGDW), Matrix Assisted Pulsed Laser Evaporation Direct Write (MAPLE DW) and Biological Laser Printing (BioLP) are methods working with this principle and are discussed within this section. One example for a different approach is stereolithography. Therein, laser light is used to induce crosslinking of the photosensitive hydrogel around the biological material instead of inducing cellular movements. Stereolithography will be introduced in Section 5.1.1 in more detail.

**Figure 1:**
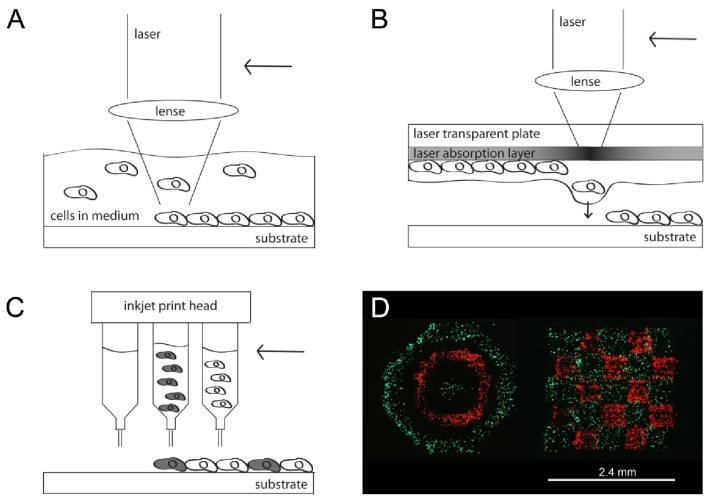
Schematic drawing of the various techniques. (**A**) During Laser-Guided Direct Write (LGDW) laser light is focused into a suspension of particles and the particles trapped by the light are pulled through the fluid and deposited on a target surface; (**B**) During MAPLE DW a laser pulse focusing on the absorbing layer evaporates the matrix containing biological material on the lower side of the substrate due to localized heating and thus pushes the material towards the substrate; (**C**) Inkjet technology ejects material piezoelectric, thermally actuated or electrostatically actuated after receiving a signal and (**D**) printed cell patterns with 2 different cell types; reprinted from [53] with permission of IEEE (© 2009 IEEE).

LGDW of living cells was developed by Odde *et al.* in 1999 [38,43]. Cells drifting by natural convection in the fluid medium were directly deposited onto an untreated glass surface by the laser. In more detail, the laser beam continuously captured cells as they drifted into the light path, pulled the cells into the center of the beam where the intensity was maximal and pushed them through the fluid medium along the beam axis onto the target surface. When the desired amount of cells, either a single cell or a number of cells, had been deposited in one spot with a spot size of 10 μm, the focusing lens was translated to move the focal point to a new spot (Figure 1A). Deposited droplets had a minimum diameter of 1 μm. A total amount of 76 cells in 26 spots with 1 to 5 cells per spot were deposited within about 30 minutes. Cells printed onto a laminin-coated fiber remain viable after 1 hour treatment. The results indicate that multiple cell types can be placed at arbitrary positions with micro-scale precision and resolutions down to 1 μm by using this technique. The terminology “direct writing” indicates that no mask or similar is used in this process, which is needed for example in photopatterning (see Section 3) [41].

Both methods, MAPLE DW and BioLP, work with the modified laser induced forward transfer (LIFT) principle [45]. During LIFT, an indirect deposition of material takes place. MAPLE DW consists of a donor substrate where the cell-containing matrix material is applied and a collector substrate at a 25–100 μm distance to which the cells are patterned (Figure 1B). On the donor side, a laser-transparent solid plate (e.g., silica glass) is coated with a laser-absorbing layer of gold, titanium or silver. On top of this laser-absorbing layer a thin liquid film of culture medium or hydrogel accommodating the bioelements to be transferred is added. A laser pulse coming from the untreated side of the plate focuses on the absorbing layer and evaporates the matrix containing biological material on the opposite side of the substrate due to localized heating. The vaporization releases the material from the support pushing it forward (“forward transfer”) [58], towards the collector slide (Figure 1B) [59,60]. More than 95% cell viability was detected on the collector slide post-transfer of the printed mammalian cells and osteoblasts. Since indirect material deposition via melting takes place, the method is not limited to photosensitive materials. A weakness of MAPLE DW is the low reproducibility and resolution of the printed cells of only 150 μm per spot [58]. This was addressed in BioLP by adding an energy conversion layer between the support and the cell layer. Experiments performed by the BioLP technique achieved spot sizes ranging from 30 μm to 70 μm, and even down to single cell resolution [47,48]. By applying the energy conversion layer the cell viability was enhanced up to almost 100%. Experiments performed with modified LIFT techniques have demonstrated no observable damage to phenotype or genotype of the printed cells [58]. Overall, laser-based methods are simple systems which work at low costs and can deposit nearly any material with micrometer-scale accuracy [38]. A major disadvantage of the system is the limited size; it can only construct small arrays of cells with a few hundred cells and deposit particles ranging from 100 nm to 10 μm in diameter [38,43].

### 2.2. Inkjet Printing

Inkjet printers are probably some of the best known devices used for cell patterning since many people use them on a daily base for printing office documents on paper. It is a drop-on-demand (DOD) material deposition technique that ejects material termed as “ink” after receiving a signal (Figure 1C). The printing process is carried out piezoelectrically, thermally actuated or electrostatically actuated, which is completely independent of the material to be ejected. Thermal inkjet printers use a heating element to generate and eject droplets through an orifice, whereas piezoelectric printers create a contraction and ejection of the fluid volume due to a voltage pulse. The print heads are specified for parallel or serial deposition with either multiple or a single head. The main requirement for applying inkjet technology for cell printing is the biocompatibility aspect. Some printers are critical in fulfilling the requirements, for example if the thermally actuated printer heats the ink up to more than 41 °C during ejection it would cause denaturation of proteins and cells.

The first approach using a modified inkjet printer for printing viable cells on a 2D substrate was performed by Boland *et al.* in 2003 [44]. They introduced a protein and cell printer made up of a commercially available HP printer body (HP 660C), modifying the system such that a glass plate can be inserted and patterned in the original paper location. Modified piezo-based HP and thermally actuated Canon print heads (BJ2200) were used by implementing temperature control in the Canon system. Cells larger than 100 μm in diameter, e.g. large mammalian cells, do not fit through commercial print heads nozzles anymore and the group had to modify the original thermally actuated HP print head with a special design consisting of independently operating piezo pumps. The ink cartridge was carefully washed and refilled with different protein and cell solutions so they could print a 2D pattern according to a Microsoft PowerPoint design. Large mammalian cells, ECs and SMCs were printed with either HP or Canon print head onto various gels such as Matrigel™ and collagen coated on the glass slide. Up to 75% of cells stayed alive and attached to their position after cell printing. Cell death was assigned to the dehydration of the cells since drop volumes were small. Both the thermally-based and piezo-based system showed potential for many applications [44]. Other groups using either thermal inkjet printing or piezo-based printing successfully deposited several neuron cells with a resolution of 85 μm [61,62], and fibroblasts, osteoblasts and chondrocytes in cell suspension printed with a nozzle of 30–60 μm in diameter [63,64]. Cui *et al.* printed ECs in drop volumes of 130 pL next to each other and could even demonstrate that the cells were able to connect and align themselves during proliferation, indicating the functionality of ECs to build tubular microvasculature [52]. High resolution printed patterns of 300 dpi (dots per inch), which corresponds to a resolution of 85 μm, were printed with a nozzle of 50 μm in diameter and imaged during 120 h in culture, showing maintenance of the design over that period [53]. In order to achieve that goal, the important role of the substrate was pointed out. Cells printed directly onto untreated slides were found to float away when culture media was added; it seems that the slide needs to be covered with a substrate conducive to cellular attachment. Some of the cells that did attach to the untreated slide could not retain their function of elongation or proliferation, which was assigned to the dry substrate. The authors stated that cells must be printed onto a wet substrate for quick attachment, but also to prevent cell stress. In their application, collagen was used as substrate, where cells attached, remained viable and kept functionality.

Laser based cell patterning methods as well as inkjet printing have been found to be promising techniques in patterning molecules and cells onto a substrate. With these techniques, viable cell patterns of various cell types in special configuration can be created and cell interactions in 2D observed (Figure 1D) [53]. A limitation for the cell patterning technique is the restriction to a 2D environment. This is a big drawback for tissue engineering especially as it has been shown that cells are strongly influenced by their 3D environment and can behave differently compared to 2D [23]. To draw conclusions on cell behavior in an environment mimicking the *in vivo* situation, 3D model systems are needed also for tissue engineering applications.

## 3. 3D Cell Patterning

**Figure 2:**
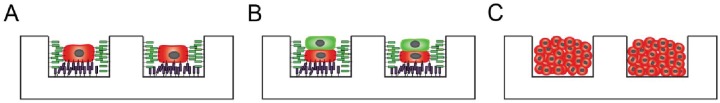
Applications of microwell array platforms. (**A**) Engineering of single cell microenvironments, mimicking the natural 3D milieu for investigating single cell behavior; (**B**) engineering of multi-cell microenvironments for investigations on cell-cell interactions; and (**C**) engineering of cellular aggregates into a microwell for investigating cell aggregate behavior. Adapted from [65] and reprinted with permission of RSC.

Besides 2D cell patterning there exists another approach where single cells are printed in a 3D environment, mostly into very small compartments in the micrometer range. This technique, which is also called 3D cell patterning, is especially used to investigate single cell, cell-cell and cell colony behavior in 3D since it provides a more physiological environment for the cells compared to 2D cell patterning. These compartments, so-called microarrays, have to be built with a defined size and geometry in which cells can eventually be trapped through gravitational forces (Figure 2) [65]. With early microarray methods used by different groups in tissue engineering [66,67], cells mainly interacted with the base of the well and not much with the sides, which then corresponds more to a 2D environment. Textor *et al.* established a microwell array system with patterns ranging from 7 to 70 μm and a spacing of 50 μm, where single cells fill the entire well and consequently experience a 3D-like environment [68,69,70]. Within the microwell, cell attachment could be enhanced by functionalizing the surface with hydrophilic fibronectin or hydrophobic lipid layers; whereas the microwell plateau was passivated to limit cell adhesion.

In another approach of 3D cell patterning Mercey *et al.* used ultra violet (UV) light for micropatterning agarose substrates [71]. This treated surface enabled the culture of single or multiple hepatocytes and HeLa cells. The results strongly indicated that this new cell array system may be suitable for high-throughput cytotoxicity and genotoxicity screening applications. A combined approach of photopatterning and electropatterning to create a local 3D microenvironment for living cell arrays was investigated by Bhatia *et al.* [41]. In their method, cells were encapsulated in a hydrogel which was filled into an assemble chamber onto a dielectric layer. Through a photomask the hydrogel-cell suspension was selectively crosslinked via photopatterning. The uncrosslinked cell suspension could be removed and replaced by a different cell solution. By applying electrical fields, dielectrophoretic forces have been used to position cells, 6 per cluster and 75 μm apart from the next cluster, within a prepolymer solution, forming cell patterns at micrometer-scale resolution where cell-cell interactions could be defined. By combining both techniques, photo- and electropatterning, a complex microstructure with various cell types as well as high-resolution cell pattern capabilities could be achieved. This provided a hierarchical control of cell positioning over length scales ranging from microns to centimeters. The group's interest was in applying fibroblasts with the help of poly(ethylene glycol)-based (PEG) hydrogel [41].

To investigate single cell-matrix, cell-cell interactions and cellular aggregates the herein mentioned methods showed quite promising results. According to need, micro-compartments of various size and geometry could be engineered and coated. Except for combined photo- and electropatterning, all techniques are limited to small scales of micrometer range and are also not made to expand to the third dimension. The photo- and electropatterning method, however, covers length scales up to centimeters. Also, the third dimension can be approached by building various layers on top of each other.Nevertheless this technique is not suitable for the investigation in whole 3D tissue constructs since for every new layer the assembly chamber has to be adapted.

## 4. Biofabrication of 3D Hydrogel Constructs Without Cells

Researchers started revolutionizing tissue engineering by first establishing 3D RP techniques for scaffold fabrication. With the layer-by-layer fabrication process it was now possible to build complex hierarchical scaffold designs [72]. Most of the methods used such as laser sintering, 3D printing and fused deposition modeling are limited to certain materials like powders or thermoplasts and thus only allow the printing of synthetic or natural scaffolds without cells [73,74,75,76,77]. It is not possible to integrate cells into the scaffold fabrication process with the mentioned techniques, since the process parameters are not physiological due to high temperature or pressure, e.g., during sintering, or contact with cytotoxic solvents such as ethanol [78]. As the scope of this review is to present methods of controlled positioning of cells in biomaterials, only scaffold fabrication methods which potentially could be used for cellular integration are considered in the following and are listed in Table 2. So far, several methods have been successfully applied for the combined placement of cells and scaffold material and the most common ones are bioplotting, inkjet printing and stereolithography. Bioplotting refers to filament printing, where as inkjet printing is known to produce droplets of the suspension. During stereolithography patterns are crosslinked via a laser.

**Table 2:** 3D biofabricated hydrogel scaffolds without cells (HA = hydroxyapatite; PEGDA = poly(ethylene glycol) diacrylate).

**Technology**	**Hydrogel**	**Fabrication specifications**	**Reference**
***Bioplotting***			
3D bioplotter Envisiontec	Gelatin, agar, fibrin, alginate	Agar: Ø Nozzle: 150 μm; pressure: 2.1 bar; deposition speed: 17 mm/s; strand diameter: 500 μm; 33 layers	[79,80]
Dispensing-based SFF technique (Asymtek)	Alginate-HA	Ø Nozzle: 330 μm; pressure: 5 bar; flow rate: 0.41 mg/s; needle speed: 5 mm/s; layer height: 300 μm; 30 layers	[81]
	Chitosan, chitosan-HA-colloidal gel	Ø Nozzle: 610 μm; pressure: 1.5–4.5 bar; flow rate: 0.0115 ml/s; needle speed: 40.7 mm/s; Ø scaffold: 610 μ μm	[82,83]
Fab@Home	Alginate	Ø Nozzle: 840μm; path width: 800 μm; path height: 710 μm; layer thickness: 300 μm; deposition rate: 10 mm/s;	[84,85]
		Ø Nozzle: 1500 μm; path width: 1650 μm; path height: 1300 μm; deposition rate: 10 mm/s	
Direct cell writing system	Alginate with iron oxide nanoparticles	Ø Nozzle: 410 μm, 250 μm; pressure: 0.3 bar, 2.8 bar	[86]
Pressure-assisted microsyringe system (PAM)	Alginate	Ø Nozzle: 20–50 μm; pressure: 0.01–0.67 bar; resolution: 0.1 μm	[87]
Direct ink writing (DIW)	Silk fibroin hydrogel	Size: 2 × 2 × 0.02 mm; Ø nozzle: 5 μm; 2–6 layers; pressure: 0.2–0.7 bar; speed: 2 mm/s	[26]
	Acrylamid -glycerol	Size: 5 × 5 mm; Ø Nozzle: 1–5μm; 1–4 layers; pressure: 0.2–0.7 bar; speed: 0.5 mm/s	[88]
***Inkjet printing***			
Inkjet bioprinter	Alginate	Microgel beads: Ø 26–40 μm; tubular structures: Ø 50–200 μm	[89,90]
***Laser-based biofabrication***			
Two-photon polymerization (2PP)	Methacrylamide-modified gelatin, PEGDA	Size: 3 × 3 × 1mm; pores: 250 × 250 μm spaced at 300 μm; feature size: 100–200 nm;	[91,92]

Landers *et al.* developed a 3D bioplotter which was able to print biological as well as non-biological solutions and hydrogels made of gelatin, agar, fibrin and alginate [79,80,93]. A multi-material open-source 3D printer called Fab@Home was developed by Cohen *et al.* [84]. They used an inverse approach by printing the chemical crosslinker calcium chloride in a bed of alginate and could show variable stiffness of the construct due to the different crosslinker concentrations. In an *in situ* study to repair an induced cartilage lesion and a bone and cartilage fracture of a cadaveric calf femur, alginate hydrogel was printed directly into the defects 5 times in a row [85]. The printed geometry closely matched the intended geometry for the chondral and osteochondral defect for all five prints per defect, with a mean error of 0 ± 200 μm for the chondral defect and 100 ± 100 μm for the osteochondral defect. In order to predict the quality of the printed scaffolds, alginate hydrogels of different concentrations have been printed and process parameters such as the deposition velocity and pressure have been varied [87]. By thoroughly investigating mechanical stiffness, rheological behavior and hydration properties a phase diagram was generated for predictions and determination of a suitable working range. Briefly, structures printed with low pressure and high velocities were well-controlled and repeatable, whereas if either pressure increased or velocity decreased the replication differed from the CAD drawing. Exceeding the critical air pressure, which was 0.04 bar in that case, only undefined structures could be realized independently of the velocity. Overall it was found that the shape of the scaffold was mainly influenced by the dynamic viscosity, velocity of deposition and pressure [87]. Stereolithography has been found to be a very strong tool in creating constructs with excellent mechanical properties for polymers such as polylactide resin [94] or polypropylene [95]. In most studies using stereolithography, cells were directly encapsulated in hydrogels without investigating the hydrogel alone. Therefore, these studies are discussed in Section 5.

A technique for printing 3D scaffolds with a very high resolution in the μm to nm range was introduced as two-photon polymerization (2PP). During the last year in particular, increasing attention was paid to this technique and a lot of research has been done by the Laser Zentrum Hannover, also by applying it to print 3D hydrogel constructs [92]. The prepolymer solution, e.g., photosensitive hydrogel, was mixed with the photoinitiator and photopolymerized via absorption at the location where the laser beam hit the sample. The absorption spectrum of the photoinitiator has to be half-wavelength of the laser radiation for the two-photon polymerization. 3D patterns can be produced by moving the laser spot according to a predefined geometry and the remaining unpolymerized material can be washed away after the process. In the study, methacrylamide-modified gelatin was mixed with 1.5 wt% photoinitiator Irgacure 2959 and photopolymerized at room temperature. The experiment demonstrated that scaffolds can be precisely fabricated with resolutions down to 100 nm using the 2PP approach. Cell compatibility was tested in a different study by seeding fibroblasts onto several 2PP fabricated PEG diacrylate (PEGDA) construct containing various photoinitiators in different concentrations [91]. The material was found to be cytotoxic when the scaffold was freshly prepared. Incubating the sample in distilled water for at least 6 days reduced the amount of the water-soluble toxic components such as the remaining photoinitiator to levels at which no cytotoxic effect was observed for the photoinitiator Irgacure 2959, whereas cytotoxicity still remained after the same amount of days for the Irgacure 369. Due to the need of aging of the photopolymerized hydrogel to reduce material toxicity to levels at which these materials are no longer cytotoxic, cells cannot be encapsulated directly into the hydrogel [91]. As soon as solutions for a novel biocompatible material will be found which also enables cell encapsulation before polymerization and guaranteeing a high cell survival, this technique might have a promising future in 3D tissue printing due to the particularly high feature resolutions.

**Figure 3:**
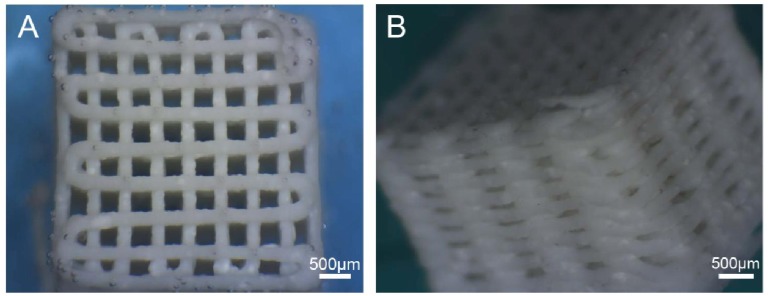
Alginate/HA scaffold after printing fabricated with a dispensing based technique. **(A)** Top view; and **(B)** side view. Reprinted from [81] with permission of IOP.

A challenge using hydrogels in biofabrication is the required adaptation of the biomaterial to the printing process. Gelation of the hydrogels has to be induced by crosslinking or polymerization and the maintenance of the 3D structural integrity during and after the assembling stage has to be guaranteed [96]. By accomplishing these requirements, gel structures can be manufactured with a controlled architecture (Figure 3) [73,81,97,98]. Once the hydrogel and its gelation method are established, it can be used as cell supporting structure by either seeding cells onto the printed construct or encapsulating cells prior to printing by assuming biofriendly fabrication conditions.

## 5. 3D Tissue Printing

Biofabrication of 3D tissue, often referred to as 3D tissue printing is the approach to print biological constructs in mm to cm size including several cell types and biomaterials at the same time [30]. Classical 3D tissue engineering methods mainly aiming to manufacture scaffolds with techniques such as salt-leaching [11], gas foaming [14] or freeze-drying [18] lack a controlled fabrication. During 2D patterning, in contrary, the system can be controlled but showed limitations concerning the missing third dimension to address research on cellular functionality. It is known that cells behave differently in 2D compared to a 3D milieu. For investigating natural cell behavior such as proliferation, migration, differentiation and drug response, 3D cellular microenvironments are advantageous.

A step to engineer whole cellular constructs in 3D was approached by Boland *et al.* [32] and Williams *et al.* [33] in 2004 as one of the first, by using stereolithographic technique and bioplotting, respectively. Compared to 3D cell patterning 3D tissue printing is not used to generate a local 3D environment for cells but an overall third dimension consisting of several layers of some mm height. Since cells need a support structure to survive and function, a suitable engineered ECM is needed until the cells produce their own ECM [99]. The latest technologies aim to combine scaffold printing and cell patterning in 3D, mainly constructing a 3D object with living cells embedded in hydrogels. With this biofabrication approach precise placement of cells throughout the construct can be achieved, including the simultaneous placements of several cell types next to each other in defined locations. The limiting conditions of the process are to maintain sterility during and after the fabrication and cell-friendly conditions such as, for example, appropriate temperature and pH range [100]. Biofabricated constructs, especially by using cell aggregates, may provide a better starting point for the cells by printing them directly on stimuli-sensitive gels and thus are faster to form functional tissue as compared to classical tissue engineering methods [101].

An introduction in bioprinting and the importance for 3D cell culture was given by Mironov [36,102,103], who also introduced the terminology biofabrication in 2009 [4]. Basically, the process can be divided into three phases [102,103]: (1) Preprocessing is for bioink preparation, including the CAD “blueprint”; (2) processing refers to the printing of the biological solution and (3) postprocessing describes the cultivation of the printed construct in a bioreactor. The postprocessing step is optional and is intended to induce maturation of the printed construct and transformation into a functional tissue. Requirements for fabricating biological tissues and organs were summarized by various groups [23,101,102,104,105]. They all agree that the printing method has to be neither toxic nor irreversibly damaging for cells and their DNA and also that the cell must retain its biological functionality. The biomaterial in which the cells are encapsulated has to be nontoxic, biocompatible, and solidify in response to specific stimuli [101]. Special flow requirements including viscosity and surface tension are needed for printing. To obtain a mechanically stable tissue right after printing the gel should provide the capacity to undergo fast and nontoxic solidification such as crosslinking [101,102]. Li *et al.* point out that scaffolds should support cell growth and tissue formation *in vitro* [104]. Concerning resolution and organization on the micro-scale there are some contradictions: Nakamura *et al.* state that biological tissues should be positioned with the highest resolution possible since they are not a random mixture of cells, but rather cells arranged to form specific micro-structures. To mimic functions and behavior of cells in 3D environments *in vitro* construction of 3D architecture and control of the inner composition is essential [23]. Mironov *et al.* however are convinced that cells have organizational capacities and by printing cellular aggregates they can organize themselves to form tissues, e.g., ECs will form tubular structures when optimal external conditions are provided [103]. In order to create a tissue of a few cm width, large amounts of cells must be handled in an appropriate manner and processed as quickly as possible [23]. In most tissues, vasculature will be indispensable since blood provides living cells with oxygen and nutrients and also removes excreted waste products for performance of their physiological functions [23,105]. An appropriate perfusion system is therefore vital to ensure cell survival and activity. The success of printing itself strongly depends on the control of the gelation state of the hydrogel layers [31].

In the past years many 3D biofabrication technologies appeared, depositing the biological material layer-by-layer to a 3D construct. The most common methods are stereolithography, 3D bioprinting, 3D bioplotting and inkjet bioprinting which will be explained in the following section. Achievements are shown through individual results and a detailed overview of the studies performed using these methods is listed in Table 4.

### 5.1. 3D Biofabrication Methods

#### 5.1.1. Stereolithography

Stereolithography is a fabrication process using a liquid photosensitive polymer that can be solidified by exposure to UV or laser light, which cures the pattern traced on the resin and adheres it to the layer below. After one layer has been solidified, the next layer of liquid polymer is applied and patterned with the laser. When a complete 3D part is formed the constructs are cleaned of excess resin and cured in a UV oven [76]. For the construction of 3D biological constructs with stereolithography, cells can be encapsulated in photopolymerizable hydrogels and gelation of the cell-hydrogel-construct can again be induced via laser or UV light. In general, the laser has the power to crosslink a special pattern within one layer depth [100]. Layer-by-layer the hydrogel-cell mixture is applied and subsequently crosslinked according to the predefined design. Several studies with different cell compatible photopolymerizable hydrogels have been performed, where the hydrogel precursor prevents the cells from damage. Boland *et al.* manufactured 3D poly(ethylene oxide) (PEO) and PEG dimethacrylate (PEGDM) hydrogels with encapsulated Chinese hamster ovary cells by laser-based photopolymerization [32]. The technique has been proven to be especially useful for the creation of complex structures which could not be built otherwise. It was also modified appropriately to make the process more biofriendly and thus enhance cell growth and cell proliferation. Bioactive PEG hydrogel precursors with encapsulated fibroblasts were exposed to a He-Cd laser to allow the formation of complex PEG structures with embedded channels of different size and orientation [106]. The group showed that 85% of the cells were viable 24 h post-fabrication. Using the photopolymerization technique photomasks can be applied additionally to selectively crosslink partial or entire layers of hydrogel precursor. Lu *et al.* set up a stereolithography system consisting of an UV light source, a digital micro-mirror masking device and a conventional computer projector which allowed to print intricate pore geometries in shape of hexagons, triangles and squares [107]. This system further allowed embedding cells in the hydrogel precursor and precisely distributing biological and chemical factors such as growth factors and ECM proteins within the structure. In one study they investigated the functionality of bone marrow stromal cells (BMSCs) which they embedded into photocrosslinkable PEG and PEGDA [107]. It turned out that especially PEGDA is an ideal polymer to encapsulate cells and sustain cell viability after photocrosslinking. Most importantly, cell functionality could be maintained; by cultivating under osteogenic conditions, cells differentiated into osteoblasts and mineralization of the ECM could be detected.

Photopolymerization has been shown to be an attractive method to crosslink hydrogel-forming polymers, resulting in mechanically strong, stable matrices suitable for cell-encapsulation. By encapsulating cells in hydrogels the viability of the embedded cells was no longer affected by the photopolymerization process, which the authors assumed was due to the lower amount of free radicals available for cell-damage [108].

#### 5.1.2. Laser-Guided Cell Patterning

Laser-Guided Direct Writing (LGDW) developed by Odde *et al.* in 1999 was introduced as a 2D cell patterning technique previously in Section 2.1 [38]. It has been used successfully in patterning cells onto a substrate and provides a strong potential to expand the technique into 3D. In order to facilitate 3D fabrication, hydrogel has to be layered on top of each deposited cell layer, followed by the next cycle of trapping cells by the laser beam and push it onto the substrate. The first 3D approach patterning ECs and hepatocytes encapsulated in collagen and Matrigel with a single cell resolution was performed by Nahmias *et al.* in 2005 [109]. In this study, three layers of cells and 500 μm thick layers of hydrogel were alternately patterned on top of each other, resulting in a true 3D pattern of cells. EC were viable to 89% after LGDW, which was not statistically different to the control without LGDW, and proliferated a few days after deposition. Hepatocytes were found to appear healthy and well-spread 24 h after guidance.

The other techniques using laser light to induce biomaterial deposition, Matrix Assisted Pulsed Laser Evaporation Direct Write (MAPLE DW) and Biological Laser Printing (BioLP), which are both working with the laser induced forward transfer (LIFT) principle have also been introduced in Section 2.1. Briefly, an indirect deposition of a material coated on the donor substrate onto the collector substrate due to local heating and evaporation of the material is performed. Chichkov *et al.* established LIFT to arrange skin cell lines and human mesenchymal stem cells (hMSCs) in well-defined patterns for research on skin regeneration and stem cell therapy [110]. The main aim of the group was to show the suitability of this technique by evaluating the influence of LIFT on cell survival, proliferation, apoptosis, DNA damage and phenotype maintenance after printing. They detected a cell survival rate of 98% and all cell types maintained their ability to proliferate after deposition. Skin cells and hMSCs did not show an increase of apoptosis or DNA damage and phenotype maintenance was proven for hMSCs. In another study the group combined LIFT with two-photon polymerization (2PP), where the PEGDA scaffolds were prefabricated with 2PP and vascular SMCs and ECs seeded by means of LIFT in a predefined pattern [111]. By combining these techniques it was possible to deposit multiple cell types precisely within the volume of the 2PP-produced scaffold. Characteristic for this study was the vertical pore orientation of the highly porous scaffold with a porosity of 90%. The results looked promising showing good control over cell density and location and a sharp transition from vSMCs to ECs. Compared to other 3D biofabrication methods, LGDW and the LIFT techniques set themselves apart by one fact: Cells are still patterned in a 2D manner onto the surface and the whole construct only becomes 3D by adding a hydrogel layer after every patterned cell layer. Cellular layers are thus separated by the hydrogel and depending on the hydrogel layer thickness the vertical cell-cell contact might be disabled. This implies that only anisotropic geometries can be generated.

#### 5.1.3. Inkjet Bioprinting

**Figure 4:**
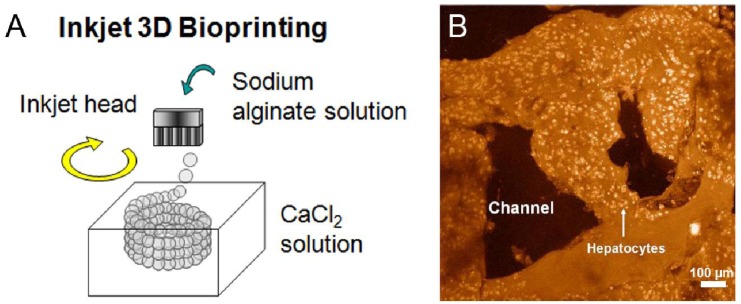
(**A**) 3D biofabrication of a tubular structure of 200 μm using inkjet technology. The homogeneously sized alginate precursor beads are deposited in circular patterns layer-by-layer into CaCl_2_ crosslinker solution forming a tubular structure due to gelation. Reprinted from [89] with permission of IS&T; (**B**) LSCM observation of a 3D bioprinted construct consisting of hepatocytes encapsulated in gelatin/chitosan hydrogel after 6 days of culture. Reprinted from [112] with permission of Elsevier.

Inkjet Bioprinting is probably one of the most promising biofabrication technologies nowadays due to its unique characteristics of high-throughput efficiency, cost effectiveness and full automation [72]. Inkjet printing is a non-contact technique which takes digital data from a computer and reproduces it layer-by-layer by depositing ink drops on previously printed successive layers. A schematic of inkjet printing is shown in Figure 4A. Thomas Boland, the pioneer of this technology, but also other groups could control cell dispensing by printing controlled volumes of liquid in defined 3D locations using a modified inkjet printer. Boland *et al.* used thermosensitive gels to generate sequential layers on top of each other to systematically approach 3D cellular assemblies with bovine aortal ECs [2]. During subsequent culture of the construct, cells have been shown to fuse within the hydrogel. Layer-by-layer printing of 5 cycles of fibrinogen, thrombin and neural cells resulted in a 3D sheet and showed that phenotypes and basic electrophysiological functions of the neurons were maintained by using the thermal inkjet printer [62]. Further, maintenance of cell viability and phenotype, differentiation capabilities as well as cell functionality after printing could be achieved for both, neurons and mesodermal cells [72].

Nakamura *et al.* applied inkjet technology to print simple cell supporting structures in shape of fibers, 2D sheets, multilayered sheets and 3D tubes [54]. Alginate hydrogel was obtained by printing the liquid hydrogel precursor sodium alginate into a calcium chloride solution, which was acting as a crosslinker to induce gelation. The authors showed in their experiments the importance of the control of viscosity on stabilizing the supporting structure. The SEA-JetTM nozzle from Epson was able to print several materials in various experiments including hydrogel precursors, living cells, growth factors and nutrients. The results showed no morphological change between cells before and after printing indicating no significant mechanical damage to living cells based on the printing procedure [55]. A limiting parameter the group had to deal with was the size of the “ink”. They were able to print structures down to 50 μm, but failed to print living cells of 10–30 μm diameters—which were too large for the print head [90].

Inkjet printing is cheap, fast and versatile, but due to the small orifice cells are exposed to high shear forces during cell printing which may cause rupture or damage to the cells [58]. Because of the small orifice size it might also not be possible to print high cell densities, which would be needed to create functional tissues [103]. Commercial print heads have nozzle diameters of less than 300 μm and can only print small drop volumes of around 0.015 μL per nozzle [44]. Various groups have been working on adapting and improving the inkjet technology to biological applications [54,113]. Such an adaptation implies prevention of droplet ejection with high temperatures and addressing problems such as cell viability and ink clogging. To increase printing speed with these small volumes of around 0.015 μL, inkjet printers generally have to be equipped with a lot more orifices than bioplotters, which can be used for printing several different cell types but also simultaneous printing of one material [58]. The maximal possible number of different inks depends on the amount of cartridges in the system. Each cartridge can be filled independently with different hydrogel precursors, cells, biological factors such as growth factors or biomaterial with different mechanical properties. Inkjet printing has been demonstrated to successfully deposit various cells including neurons, ECs, SMCs and HeLa cells in 3D [54,62,113]. It has been shown to be fast and with a nozzle size of around 30 μm to 200 μm high resolution of about 85 μm to 300 μm could be achieved [36,62].

#### 5.1.4. Bioplotting

One of the newer techniques is 3D bioplotting which is a biofabrication technology based on the extrusion of continuous filaments. It is sometimes also referred to as direct write system, compared to drop-based deposition in inkjet technology. During bioplotting, the ink is stored in a syringe or a similar device and plotted in filaments spatially controlled by an X-Y-Z robotic system [36]. One of the first devices for biofunctional and cell compatible printing using syringe-based material deposition was developed at the Freiburg Materials Research Center in 2002 under the guidance of Rüdiger Landers and Rolf Mülhaupt [79] and in cooperation with Envision Technologies (Envisiontec GmbH, Gladbeck, Germany). While Landers *et al.* mainly used the bioplotter to investigate hydrogel scaffolds without cells, Fedorovich *et al.* used the same bioplotter including cells in their material for approaches towards bone tissue engineering in 2008 [30]. BMSCs were mixed with different hydrogel precursors such as Lutrol F127, agarose, alginate and methylcellulose solutions and printed with the bioplotter device. Identical deposition speed and needle diameter for printing the various materials resulted in different fiber diameters and scaffold architectures due to the different gelation rates of the hydrogels. Agarose had a slow gelation rate and thus resulted in broad fibers which might fuse under their own weight, compared to the fast gelation of alginate. Lutrol F127 deposition could be easily controlled and various fiber configurations up to 10 layers were achieved. No difference in cell survival was detected for the printed and the unprinted control group, but differences in cell survival could be observed depending on the hydrogel used. BMSC viability of only 4% was detected in Lutrol constructs after 3 days compared to over 90% cell viability in alginate and Matrigel. BMSC viability in agarose was significantly lower than in alginate and Matrigel and was down to 65% 3 days post-printing. Furthermore, it was observed that BMSCs in alginate spread homogenously inside the gel and retained their ability to differentiate along the osteogenic lineage after extrusion. In a subsequent study the researchers embedded BMSCs in Lutrol hydrogel, added the photoinitiator and photopolymerized the rectangular construct of 20 × 20 mm after printing 10 layers with a single layer thickness of 150 μm [34]. During photopolymerization, UV light split photoinitiator molecules into radicals initiating the formation of a polymer network which resulted in a stable, mechanically strong structure. BMSCs remained viable and maintained their ability to differentiate towards the osteogenic lineage post-printing and post-photopolymerization. Cell viability was significantly higher in the photopolymerized construct with more than 70% viable cells after 1 day compared to the unphotopolymerized control group with around 45% viability.

Another bioplotter called BioAssembly Tool (BAT) was developed in 2004 by the company Sciperio, Inc (Stilwater, OK, USA) and was able to print biomaterials, biomolecules and cells with a wide range of viscosities. Smith *et al.* performed a validation study using BAT where BAEC were encapsulated in collagen and fibroblasts in Lutrol F127. BAEC survival was between 46% and 86% depending on the tip diameter, being 200 μm and 500 μm, respectively. The results showed that the smaller the tip the less cell survival there was. Printed line width was up to almost twice the tip diameter, resulting in 800 μm width for the 500 μm tip and 200 μm width for the 200 μm tip, with a deviation of printed line width of only 9% [33].

A robotic printing platform called Fab@Home was developed by Lipson *et al.* in 2006 with the idea to provide a universal platform for 3D fabrication [114]. The device had to be comparably cheap and accessible for a large group of users with different backgrounds and from different fields. During the first attempts various materials such as silicon, epoxy and chocolate was printed. Tissue engineering was only one application of Fab@Home, where the authors printed encapsulated chondrocytes in alginate hydrogel precursor after pre-crosslinking with calcium sulfate [115]. Viability tests after printing showed the successful application of Fab@Home in tissue engineering with a cell survival of 94% and a homogenous cell distribution.

Wang *et al.* built their own 3D syringe-based bioplotter and investigated its feasibility for a number of different cell types in several hydrogels [28,112]. Engineering an artificial liver was first performed by printing hepatocytes encapsulated in gelatin/chitosan and gelatin hydrogel precursors. An advanced approach was followed by printing adipose-derived stromal cells (ADSCs) embedded in gelatin/alginate/fibrinogen hydrogel precursor to form a vascular-like network next to hepatocytes in gelatin/alginate/chitosan [116]. The group could show that ADSCs were successfully induced into ECs, but only formed spindle shapes, a major characteristic of ECs, at the periphery of the strands. The authors suggested this outcome might have been due to the short culture time in the 3D construct. An example of a 3D bioprinted construct consisting of hepatocytes and gelatin/chitosan is shown (Figure 4B).

Sun *et al.* built a home-made multi-nozzle biofabrication system [117]. Four types of nozzles were implemented in this system ranging from 30 μm to 500 μm in diameter, with each of the nozzles actuated by another mechanism: solenoid-based, piezoelectric, pneumatic or by spraying. This variety of possibilities allowed the group to simultaneously deposit cells, growth factors, scaffold materials and other bioactive compounds. ECs were encapsulated and printed in alginate hydrogel precursors with an accuracy of 10 μm. The performed cell experiments showed that cells survived in culture more than 21 days post-printing and proliferated to a cell number which is 6 times higher than the original cell number. The results were used to validate their mathematical model for predicting flow rate of the ink, the printed filament diameter and final scaffold porosity. Recently the group investigated a new hybrid nanobioprinting technique, which combines the initial patterning capabilities of syringe-based cell deposition with the active patterning capabilities of super paramagnetic nanoparticles [118]. The authors found that if paramagnetic nanoparticles were in the alginate no impact on cell viability was detected directly after printing. Nevertheless, a loss in cell viability of 16% or 35% for a nanoparticle concentration of 0.1 mg/mL or 1.0 mg/mL, respectively, was found 36 h post-printing. Loading nanoparticles in cells already decreased cell viability to 11% which was further decreased by 29% 36 h after printing. The group showed that nanoparticles or magnetically labeled cells could be moved with a magnet in a low viscous alginate gel depending on the magnet intensity.

A dispensing tool from nScrypt (Orlando, FL) was used by Jakab *et al.* to look into the self-organizing capacity of cells and tissue to construct functional living structures of prescribed shape [31]. The idea was to mimic early structure-forming process in the embryo such as the liquid-like behavior of tissues for bio-ink fusion. For this approach the researchers used their bioprinter in several phases of the fabrication process: They first extruded a cellular “sausage” consistent of embryonic cardiac cells and ECs with their printer and cut it into equal-size cylinders. These multicellular cylinders were incubated overnight rounding into spheroids. The spheroids were applied again to the bio-ink cartridge and delivered into a preprinted polymerized hydrogel including vascular endothelial growth factor (VEGF). The spheroids placed next to each other on the hydrogel fused after 60 h and it could be detected that ECs organized into vessel-like structures. This approach is also called scaffold-free tissue engineering, describing the direct printing of highly concentrated cell spheroids in a predefined location without a scaffold that provides a 3D structure. The group printed these spheroids into 3D constructs layer-by-layer in a later study [119,120].

### 5.2. Comparison between the Different Biofabrication Techniques

Comparing the various techniques among each other it can be stated that inkjet printing is the least technologically intricate and least expensive method of all cell printers and has the advantage of a non-contact printing technology [23]. It has been shown to be fast and with nozzle sizes between 30 μm to 200 μm high levels of resolution of about 85 μm to 300 μm could be achieved [36,62]. Due to the small orifice high shear forces appear during cell printing which may cause rupture or damage to the cells [58]. The harsh printing conditions are still a technical barrier in printer development for delivering viable cells; the cell viability decreases significantly especially for larger constructs which need longer printing time [58,72]. Despite these restrictions former experiments using thermal and piezo-based inkjets successfully printed living cells [2,44,58,64]. LIFT and modified LIFT technologies are the only nozzle and orifice-free techniques that are capable of printing living cells. The advantage of this setup is that there is no risk of clogging and thus cells with high cell densities of 108 cells/mL as well as viscous fluids can be printed with resolutions of 10 μm to 100 μm, still guaranteeing a very high cell viability of 95–100%. Concerning the impact of cells on substrates during the printing procedure, inkjet as well as modified LIFT techniques showed similar droplet velocities of 5–20 m/s and similar impact forces [58]. Although these forces may seem detrimental to cells and biological agents such as growth factors, the high cell viability achieved in studies using those techniques demonstrates the potential of these techniques. LGDW works based on high resolution cell deposition [58]. With this technique single cells can be trapped in the laser and deposited onto a surface, which allows a precision in the micrometer range. Due to the tiny amounts of biological solutions needed, only small 3D-like constructs consisting of one single cell type can be built up [36]. Stereolithography was one of the first techniques in layer-by-layer manufacturing providing a comparably high layer resolution of up to 1.3 μm and a laser spot size of 80–125 μm [76]. By using this technique, only photosensitive hydrogel precursor can be addressed, which results in a limited choice of materials since these hydrogels have to be biocompatible for cell encapsulation. Examples for suitable materials are PEG and derivatives, PVA, PEO and modified polysaccharides such as hyaluronic acid and dextran methacrylate [76]. The key points highlighting the main differences of the various biofabrication techniques are summarized in Table 3.

**Table 3:** Key points of the various 3D biofabrication techniques.

**Technique**	**Stereolithography**	**LIFT/LGDW**	**Inkjet**	**Bioplotting**
*Resolution*	1.3 μm	10–100 μm	85–300 μm	Some 100 μm
*Load volume*	nL–mL range	>500 nL	mL range	mL range
*Scale-up*	Depending on the stereolithographic	Not very suitable due to stacking of 2D layers	Multiple print heads	Very suitable, no geometrical limits
*Element size*	80–125 μm	<100 μm	30–200 μm	Some 100 μm
*Pros*	Very high printing resolution possible	High cell densities possible	Best known and cheapest technique	Printing of large constructs in cm range possible
*Cons*	Only photosensitive hydrogels possible	Not suitable for 3D constructs in the mm range	High shear forces; clogging	Lowest printing resolution of several 100 μm compared to other methods

It would be wrong to consider these methods as competing technologies. Depending on their biological application, one or the other bioprinting method is more suitable [102,121]. An important parameter is certainly the resolution of the different techniques. Being interested in biological cues, high resolution prints are needed, however, the object size does not play a role and small 3D objects of a few μm widths are sufficient. LGDW provides high resolution prints and thus is an adequate method to investigate cell-cell interactions or self-assembly studies [109]. Another factor which plays a role in choosing the right technique for a defined application is the load volume of the particular printers. Syringe-based deposition systems as well as inkjet based systems allow large amounts of cell-hydrogel mixture in the mL range, with the drawback that a certain amount of the media always remains in the cartridge or syringe. Modified LIFT techniques, in contrast, work with small amounts down to 500 nL load volume, allowing cells to be deposited in ultra-small quantities. This can be of great advantage if, for example, only very small amounts of cells are available; on the other hand it is not very convenient when larger cellular constructs are desired [47,58]. If the focus is on creating larger tissue constructs with a few millimeter lengths, bioplotting technology will be the method of choice since sufficient amounts of biomaterial can be deposited in a reasonable time. Scale-up of the presented methods would be important if one is interested in building 3D constructs. The scale-up is needed in terms of geometrical size, the number of different cell types within one setup, but also the increase in printing speed with parallel printing to reduce the process time. LIFT and LGDW techniques are probably not very suitable for scale-up since cells are patterned in a 2D manner onto the surface and the whole construct only becomes 3D by adding a hydrogel layer after every patterned cell layer. For each layer the hardware configuration has to be modified.

#### 5.2.1. Modular Tissue Engineering

Modular tissue engineering addresses the challenge of recreating biomimetic structures by designing tissue on the microscale that can be used as building blocks for creating larger tissues. To create these modular tissue units, multiple methods such as cell aggregation, microfabrication of cell-loaded hydrogels or cell printing can be used. In this so-called “bottom-up” approach, these blocks are eventually assembled into tissues with specific features through stacking of layers [122] or direct assembly [123], to name some of the methods. Many tissues are composed of repeating functional units e.g., the liver lobule and for those tissues the bottom-up approach seems to have a strong biological basis [124,125]. Modular tissue engineering aims to create more biomimetic engineered tissues by mimicking the native functional units.

**Table 4:** Biofabrication of 3D cell-hydrogel constructs (LGDW = Laser-Guided Direct Writing; BioLP = Biological Laser Printing; LIFT = Laser Induced Forward Transfer; 2PP = Two-Photon Polymerization; BAT = BioAssembly Tool; ECs = endothelial cells; hMSCs = human mesenchymal stem cells; SMCs = smooth muscle cells; BMSCs = bone marrow stromal cells; ADSCs = adipose-derived stromal cells; PEG = poly(ethylene glycol); DM = dimethacrylate; DA = diacrylate).

**Technology**	**Hydrogel**	**Cells**	**Fabrication specifications**	**References**
***Stereolithography***				
	PEG	Fibroblasts	Laser beam Ø ∼250 μm; layer thickness: ∼250 μm	[106]
	PEO, PEGDM	Ovary cells	Ring scaffolds: Ø: 5.3 mm; thickness: 1.5 mm; UV laser spot: 250 μm; resolution per layer: 150 μm; x–y resolution: 250 μm	[32]
***Laser-based biofabrication***			
LGDW	Collagen, Matrigel	ECs, hepatocytes	Single cell resolution	[109]
BioLP	Matrigel	Osteosacroma cells	2 layers of cells separated by a 75 μm layer of hydrogel	[49]
LIFT	PEGDA, Alginate, EDTA, blood plasma, Matrigel; Collector slide: agarose	Fibroblasts/keratino cytes, hMSCs, ECs	Ø Droplets: 80–140 μm; speed: 1200 cell droplets/min; scaffold height including 6 layers: 300 μm; focal spot: 45 μm; distance between spots: 75 μm; accuracy: 5 μm	[60, 110,111]
LIFT-2PP	PEGDA	SMCs, ECs	Ø laser spot: 45 μm; laser transferred droplet size: 80–140 μm	[111]
***Inkjet printing***				
Inkjet bioprinter	Fibrin gel (fibrinogen + thrombin)	Neural cells	25 orifices with Ø 50 μm, resolution: 85 μm; 250,000 drops/s; 5 layers	[62]
	Alginate, fibrinogen, thrombin	HeLa cells, ECs	Speed: 20 mm/s; ejection time: 800 Hz; pattern Ø: 1 mm; size: 5 × 7 mm	[54,99]
	Collagen	SMCs	Size of construct: 5 × 5 mm, 5 layers; thickness per layer: 16.2 μm;	[113]
	Fibrin gel (fibrinogen + thrombin)	ECs	Size: 10 × 5 × 2 mm; 2 layers	[126]
***3D bioplotting***				
Bioplotter Envisiontec	Alginate, Lutrol F127, Matrigel, agarose, methylcellulose	BMSCs	Ø Nozzle: 100–400 μm; 4–10 layers; thickness per layer: 150 μm; spacing 300 μm; speed: 1–30 mm/s; pressure: 0.5–3 bar; size: 20 × 20 mm	[30,34]
BAT	Polyoxyethylene– polyoxypropylene collagen I	Fibroblasts, ECs	Ø Nozzle: 200–500 μm; resolution ≤ 5 μm; accuracy ≤ 5 μm; deposition rate: 12 nL/s–1 mL/s; speed: 10 μm/s–50 mm/s; size: 2 × 2 × 1.5 mm; layer height: 50–100 μμm; pressure: 1.2 bar	[33]
Fab@Home	Alginate	Chondrocytes	Ø Nozzle: 840 μm, nozzle precision: 25 μm, width: 1200 μm; height: 800 μm; flow rate: 0.6 mL/s; size: Ø: 6 mm × 2 mm height	[115]
	Methacrylated hyaluronic acid, methacrylated ethanolamide, PEGs	Hepatoma cells, epithelial cells, fibroblasts		[127,128]
Bioplotter	Gelatin/chitosan	Hepatocytes,	Ø Nozzle: 300 μm; drop volume: 20 nL; lateral resolution: 10 μm; X-Y velocity: 10 mm/s; extruding velocity 30 mm/min; pressure: 0.3 bar; layer height: 180 μm	[28,112]
	Gelatin/alginate elatin/alginate/fiinogen elatin/alginate/chosan	Neuron cells; br Schwann cells, ADSC, hepatocyte it	Ø Nozzle: 250μm, X-Y velocity: 5 mm/s; extruding velocity 15 s mm/min; layer height: 150 μm; width: 380 μm	[116,129]
Multi-nozzle SFF deposition system	Alginate	ECs, fibroblasts, hepatocytes	Droplet-based or continuous deposition; Ø Nozzle: 30–500 μm; velocity: 10 mm/s; pressure: 0.3–2.8 bar; 40 layers	[117,130,131,132,133]
Cell writing system	Alginate with iron oxide nanoparticles	ECs	Size of construct: 5 × 5 × 2 mm Printing pressure: 0.3 bar	[118]
Dispensing-based deposition system	Mebiol (N-isopropylamid and poly oxyethylene)	Insect cells	Feed speed: 0.5–0.83 mm/s; pressure: 0.3–0.4 bar; line width: 114–300 μm; size: 1 × 1 mm	[134]
nScrypt bioprinter	Collagen I, agarose	Embryonic cardiac cells, ECs, ovary cells, SMCs, fibroblasts	Tubes: Ø 900–25,000 μm, wall thickness 300μm	[31,119]

#### 5.2.2. Organ Printing

Organ printing refers to self-assembly of cellular aggregates which are placed by any of the bioprinting technologies [4,101,135]. The focus in organ printing lies on using a biofabrication device for placing the whole prefabricated cellular aggregates, whereas in modular tissue engineering the small building units are printed by a biofabrication device and further assembly can be done by any method. Cellular aggregates can be one way for building the small units in modular tissue engineering, whereas organ printing only refers to the application of cellular aggregates. The idea in organ printing is to employ cell aggregates which matured to tissue spheroids as building blocks and let them fuse after deposition. Self-assembly-based tissue engineering uses the organizational capacity of cells to build tissues and eventually organs by mimicking natural morphogenesis. For example, ECs are genetically predestined to form blood vessels and will form tubular structures on their own when the right external conditions are provided. The hypothesis is that by providing the correct external conditions—which remains quite a challenging task—there should be no need to pre-shape a scaffold to obtain a certain structure. To achieve fusion and structure formation of the multicellular aggregates, a gel with optimal properties has to be designed. Fusion could be correlated to tissue liquidity and the similarity between liquids and tissues composed of motile and adhesive cells [103]. If the aggregate was too cohesive cells could not migrate, whereas if the aggregate was not sufficiently cohesive, cells dispersed into the gel. In either case no fusion took place [101]. Using cellular aggregates composed of thousands of cells, the processing time was significantly reduced since a large number of cells can be printed at once. This also led to enhanced cell survival, since less individual cells within cellular aggregates experienced the harsh printing conditions by passing the nozzle but also due to the shorter overall printing time [101]. Another advantage of organ printing is the high control level of cell and ECM placement for various biological materials that can be positioned simultaneously. Organ printing also offers the possibility to approach the problem of vascularization in thick tissue constructs, e.g. by controlling the release of growth factors such as VEGF or by incorporating a branched vascular tree such as in the approach of Mironov *et al.*, which is discussed in detail in Section 5.2.3 [101,103,136].A drawback of this technique is that the structural support is dependent on the self-assembly process and is therefore less controllable [100]. Tissue engineering by self-assembly of scaffoldless printed cellular units has been discussed by Jakab *et al.* [120]. Recent overviews of trends and challenges in organ manufacturing focus on the importance of the hydrogels for cell organization and the fabrication of vascular systems [105,136].

#### 5.2.3. Biofabrication Facing the Vascularization Problem

In tissue engineering vascularization of larger 3D constructs is still one of the most critical points since without sufficient perfusion, nutrient supply and waste removal cannot be guaranteed and thus cells cannot survive or function like in normal tissues or organs [36,137]. In particular, more complex vascularized organs like for example heart, liver or kidney can only be effectively perfused with a network of vascular structures. This so-called intraorgan vascular tree has to be composed of large vessels correlating to arteries and veins in mm to cm size, intermediate vessels simulating small arteries and veins as well as arterioles and venules in the μm to mm range, and small vessels mimicking capillaries in the μm range [101]. The easiest approach to design perfusion is to create channels into 3D scaffolds or cellular constructs (Figure 4B). However, during culture the construct will change due to cells proliferating, differentiating or producing ECM and also due to the disintegration of the cell supportive matrix. Due to this natural process, the channel structures deteriorate and thus constrain the medium supply. By using biofabrication techniques, vessel inducing cells such as ECs were applied on defined locations mimicking real vessels and their function [52]. ECs have been shown to form tubular structures and integrity after 21 days of culture. Up to now the engineered vessels are mainly used to perfuse the construct with cell culture medium, compared to *in vivo* where the supply of nutrients and the removal of waste products take place via blood-flow. Jakab *et al.* described the potential to connect the printed vascular tree after *in vitro* maturation in a bioreactor to an *in vivo* host vasculature [120]. It has been shown that by using bioprinting technology to address this challenge, the first successful results for engineering vessels were obtained [52,120,138].

## 6. Future Directions

3D biofabrication is a vibrant research area for which very promising first results have been obtained in the last decade and which has the potential to grow tremendously within the next few years. It will find applications in organ printing for *in vivo* substitutes, tissue reconstructions for *in vitro* biotechnological and pharmacological needs such as drug-testing where animal testing may be partially replaced by use of 3D biofabricated tissue [99]. Drugs—either by themselves or embedded in microspheres or nanospheres—could be deposited within the construct optimizing the tissue formation. Researchers have used inkjet printing technology for cell-based gene therapies [126]. They showed that it is possible to transfect genes into cells by inkjet printing besides the precise delivery of the modified cells to a given target. Within the next few years many new niches for RP tools will arise in order to meet the needs of the specific applications. Digital bioprinting might be one example where the biomaterial is labeled with magnetic nanoparticles and can be positioned in a controlled manner due to magnetic interactions [4]. Mironov's prediction for cell printers is that they will be as common for biological academic, clinical and industrial laboratories as microscopes are today [36].

Most of the published results up to now have been proof-of-concept studies where it has been shown that cell deposition encapsulated in various hydrogels is possible. Among these, only few studies have investigated process parameters either for predictions or optimization strategies in a systematic way. One validation study for quality predictions of the printed scaffolds was performed by Tirella *et al.* who printed alginate hydrogels of different concentrations by varying process parameters such as the deposition velocity and pressure [87]. The resulting phase diagram could be used for predictions and determination of a suitable working range. Sun *et al.* investigated cell viability and cell recovery after printing as a function of dispensing pressure and nozzle diameter [133]. Cell viability and cell recovery increased with decreasing dispensing pressure and/or increased nozzle diameter; with the majority of cell recovery occurring within the first 24 h. Especially for reproducibility it is important to define a suitable working range keeping variations to a minimum. To establish the biofabrication technology for a broad range of applications including different materials and feature sizes such as the diameter of the deposited filament or droplet, a lot of validation work on quality and reproducibility still needs to be done.

Stem cells might play an important role for 3D tissue construction because they provide high potential for the creation of complex constructs, which has been already highlighted recently by Mironov: “Stem cell biology is a potentially important component for biofabrication because stem cells are the main raw materials for tissue bioassembly” [4]. A challenge in using stem cells for biofabrication applications will be to optimize the cellular environment such as the hydrogel; combining the advantages of cell attachment, cell stimulation and mechanical stability to mimic the *in vivo* environment to the highest degree. Modifications of mechanical and intrinsic properties and protein as well as growth factor delivery have to be investigated in more detail. Stem cell regulation and the maintenance of a specific phenotype depend on various biological factors such as growth factors but also matrix elasticity, which has been shown in previous studies [6,7,139,140].

Mathematical modeling and computer simulations are important tools in modern fabrication schemes to optimize process design and for predictions [4,137]. 3D biomaterial deposition has been studied by Chen *et al.* since it has been shown that inappropriate printing parameters like the deposition speed can dramatically influence the outcome [82]. The model included process parameters, structural parameters and flow behavior. Experiments to print chitosan scaffolds with a dispensing-based RP technique were performed to validate the model. Results showed a good agreement with the model predictions and also further studies with alginate-hydroxyapatite hydrogels corroborated the mathematical model [81]. Monte Carlo simulations were performed assigning liquid-like properties to the tissue and gel-tissue interfacial tension as control parameter [141]. In this study nice agreements between experimental results and simulation was found. Sun *et al.* performed a lot of research on computer-aided tissue engineering, the application of computer-aided technology combined with biology in tissue engineering. CAD, image processing, manufacturing and SFF for modeling, designing, simulation and manufacturing of biological tissue and organ substitutes has been performed [142]. *In silico* performance of cell fusion is becoming a focus of interest also to optimize process conditions and the final outcome. Forgacs *et al.* investigated mathematical models for predicting post-printing tissue remodeling, supporting the idea that tissue liquidity may provide a mechanism for *in vitro* organ building [143]. Using computer simulations, the constructs can be predicted and hence optimized before printing. Nevertheless, this approach will need more attention to establish it as common tool for designing new biological processes and 3D tissues.

## 7. Discussion and Conclusions

In the last decade an increasing number of researchers have been investigating new methods for biofabricating 3D cellular constructs using complex designs. The amount of publications and the creation of new societies indicate the strong growth potential of this new field [137]. These techniques are still in their infancy, but nevertheless first successes could already be recorded. 3D biofabrication has been shown to be suitable for the manufacture of desired scaffold geometries with different materials. It further has the potential to provide a controlled placement of viable cells. The impact of fabrication conditions on cell viability and scaffold materials, a key aspect for new tissue generation, have already been investigated thoroughly. If attention was paid to physiological printing conditions such as temperatures below cell denaturation and the application of nontoxic gelling methods, several devices showed no impact on cell viability [30,118]. Cell functionality was examined in various studies by observing, for example, tube formation of ECs [52] or osteogenic differentiation of BMSCs via matrix mineralization [107]. This is important since biofabrication methods will only have a future when no impairment of cellular function during processing can be guaranteed. Cell viability, phenotype maintenance, differentiation capabilities as well as cell functionality were found to be stable for those printing techniques mentioned in this review when appropriate conditions were applied [30,72].

In theory, biofabrication techniques should be able to recapitulate living structures down to very small scales [40]. It has been shown that it is not possible, at least for the moment, to achieve such detailed results. Recently, investigators looking into cell fusion and self-assembly came up with a new concept which might mitigate the need to reconstruct tissues down to a cellular level. They found that it was not necessary to produce precise replicas of biological organs, if cells were provided with the adequate environment and signals and that if those requirements are met, the cells will assemble according to their natural function. Scales from meter to micrometer range could be designed and fabricated by 3D bioprinting devices and the microarchitecture in nanoscale range could then be formed by cells and the surrounding material [144]. In order to guarantee cell self-assembly, the devices need to be able to print cells as closely together as possible so they can merge and form cellular aggregates. Results revealed that cells in a hydrogel-rich and cell-poor construct remained viable but rarely connected to form tissues [105]. Hydrogels have been shown to be promising materials for creating a suitable environment for cells providing adequate strength [23,35]. They can be designed according to a predefined geometry and thus it is possible to influence porosity within the construct. Mechanical properties of the cell-hydrogel construct can be modified, for example, due to porosity adaptation without changing the basic material [131].

Overall it can be said that the biofabrication methods described in this review are very suitable for cell placement and scaffold fabrication according to a predefined design; but the various technologies are still in their infancy and have to be improved. Especially in terms of resolution, repeatability and precision of cell placement, much research still needs to be done in the future. One of the most critical points to address is the need of down-scaling to enable single cell placement but to avoid clogging or drying of the cells at the same time. Especially for the construction of larger constructs, printing on the micro-scale is not feasible yet in reasonable time.

This highly interdisciplinary research area is still in its infancy. To address the very complex tasks specialists of various expertises in tissue engineering and biology, mechanical engineering as well as material science is needed. The interdisciplinary groups have to approach each other, updating and combining their current state of research and knowledge in order to achieve optimal results. The idea of this review was to introduce or update tissue engineers and biologists to the new technical opportunities available today to reduce comprehension barriers and to demonstrate the potential of this field.
